# Stimulation of the Internal Ribosome Entry Site (IRES)-Dependent Translation of Enterovirus 71 by DDX3X RNA Helicase and Viral 2A and 3C Proteases

**DOI:** 10.3389/fmicb.2018.01324

**Published:** 2018-06-19

**Authors:** Yu-Siang Su, Ai-Hsuan Tsai, Yueh-Feng Ho, Shin-Yi Huang, Yen-Chun Liu, Lih-Hwa Hwang

**Affiliations:** Institute of Microbiology and Immunology, National Yang-Ming University, Taipei, Taiwan

**Keywords:** enterovirus, internal ribosome entry site (IRES), DDX3X, RNA helicase, viral protease

## Abstract

The translation of enterovirus 71 (EV71) is mediated by an internal ribosome entry site (IRES)-dependent manner. EV71 IRES comprises five highly structured domains (domains II-VI) in the 5′-untranslated region of the viral mRNA. A conserved AUG triplet residing in domain VI is proposed to be the ribosome entry site. It is thus envisaged that the highly structured conformation of domain VI may actually reduce the accessibility of the AUG triplet to the ribosome. This study identified a DEAD-box family RNA helicase, DDX3X, that positively regulated the EV71 IRES-dependent translation. The helicase activity of DDX3X was required for the stimulation of EV71 IRES activity; however, DDX3X was no longer important for the IRES activity when the secondary structure of domain VI was destabilized. DDX3X interacted with the truncated eIF4G which bound specifically to domain V. Thus, we proposed that DDX3X might bind to domain VI or a region nearby *via* the interaction with the truncated eIF4G, and subsequently unwound the secondary structure of domain VI to facilitate ribosome entry. Additionally, we demonstrated that the viral 2A^pro^ and 3C^pro^ enhanced the IRES-dependent translation *via* their protease activities. Together, these results indicate that DDX3X is an important RNA helicase involved in EV71 IRES-dependent translation and that IRES translation is enhanced by viral infection, partly mediated by viral protease activity.

## Introduction

Enterovirus 71 (EV71) infection causes hand-foot-and-mouth disease (HFMD) mainly in infants and young children. In most cases, EV71 infection is self-limiting, but occasionally it causes neurological complications, such as encephalitis, aseptic meningitis, and acute flaccid paralysis, or can even lead to death (McMinn, 2002). EV71 belongs to the *Picornaviridae* family. The EV71 genome encodes a large polyprotein that is further processed into 11 structural and non-structural proteins. The 5′-untranslated region (5′-UTR) of EV71 is approximately 750 nucleotides (nt) that contains a cloverleaf structure and an internal ribosome entry site (IRES). The cloverleaf structure (domain I) is an important *cis*-element for viral RNA replication (Barton et al., 2001; Lyons et al., 2001), and the IRES element (domains II-VI) directs the initiation of translation of the viral mRNA in a cap-independent manner (Thompson and Sarnow, 2003).

EV71 IRES spans approximately 450 nt. A Yn-Xm-AUG motif, conserved in most of Picornaviruses, is located at the 3′ border of the IRES, in which Yn (a pyrimidine tract, *n* = 8–10 nt) is separated from an AUG triplet by a spacer Xm (*m* = 18–20 nt). This AUG triplet in EV71 is located at nt 592–594, which is sequestered within domain VI and remains silent in most of the time. However, the AUG_592_ triplet has been previously proposed to serve as the “ribosome landing pad,” which is recognized by the 43S ribosome complex but not used as the translation initiation codon (Nicholson et al., 1991; Pilipenko et al., 1992). Instead, the assembled 48S complex initiates translation approximately 150 nt downstream at the AUG_746_ triplet.

An enteroviral infection shuts off cap-dependent translation *via* the cleavage of eIF4G and polyA-binding protein (PABP) (Lloyd et al., 1987; Lamphear et al., 1993; Joachims et al., 1999; Bonderoff et al., 2008) as well as the phosphorylation of eIF2α (Walsh and Mohr, 2011) to facilitate IRES-dependent translation. Nevertheless, the IRES-mediated translation still requires canonical initiation factors such as the C-terminal cleavage fragment of eIF4G, a product of viral protease. A recent *in vitro* reconstitution experiment further demonstrated that the translation of poliovirus (PV) or EV71 IRES required several other canonical initiation factors, such as eIF4A, eIF4B, eIF3, eIF2, and eIF1A (Sweeney et al., 2014). The truncated eIF4G binds specifically to domain V and recruits eIF4A. The binding of eIF4G and eIF4A to domain V induces conformational changes downstream of their binding site near the 3′-border of IRES, which are thought to facilitate the recruitment of the 43S ribosome complex (de Breyne et al., 2009). However, the factors that mediate these conformational changes have remained unclear.

In addition to the canonical factors, several cellular proteins, known as IRES *trans*-activating factors (ITAFs), were reported to modulate IRES activity. These include poly(rC) binding protein 1, 2 (PCBP1, 2), polypyrimidine tract binding protein (PTB), glycyl-tRNA synthetase (GARS), the lupus antigen (La), SRp20, upstream of N-Ras (Unr), far upstream element binding protein 1 (FBP1), HuR/Argonaute 2 (Ago2), and nuclear protein Sam68, among others (Hellen et al., 1993; Meerovitch et al., 1993; Blyn et al., 1996; Hunt et al., 1999; Choi et al., 2004; Bedard et al., 2007; Huang et al., 2011; Andreev et al., 2012; Lin et al., 2015; Zhang et al., 2015). Interestingly, many of these ITAFs are associated in ribonucleoprotein complexes that were originally located in the nucleus but are translocated to the cytoplasm during Picornaviral infection (Lloyd, 2015). With the assistance of these ITAFs, IRES-dependent translation can continue or even be enhanced when cap-dependent translation is repressed during a viral infection.

In addition to the eIF4A RNA helicase, in this study we have identified another RNA helicase, DDX3X, which is also important for EV71 IRES-mediated translation. DDX3X is a DEAD-box RNA helicase that shuttles between the nucleus and the cytoplasm and has RNA-dependent ATPase/helicase activity (Rocak and Linder, 2004; Schroder, 2010). DDX3X is a multifunctional protein that has been implicated in transcriptional and post-transcriptional regulation, RNA splicing, RNA migration, and the initiation of translation (Schroder, 2010). The role of DDX3X in mRNA translation is controversial. While some data suggest that DDX3X acts as a suppressor of the initiation of translation *via* binding to eIF4E (Shih et al., 2008), several other reports indicate that DDX3X acts as a supportive translational factor (Lee et al., 2008; Lai et al., 2010; Geissler et al., 2012). It has been shown that DDX3X is specifically required for several cellular and HIV transcripts that harbor RNA secondary structures at their 5′ ends. These structures are stable enough to resist the unwinding activity of eIF4A at translation initiation sites (Lai et al., 2010; Soto-Rifo et al., 2012). DDX3X, *via* its interaction with eIF4G and PABP, can bind to the 5′ ends of these target mRNAs, where it cooperates with eIF4A to destabilize the secondary structure and facilitate ribosome entry (Soto-Rifo et al., 2012).

In this study, we presented data demonstrating that DDX3X stimulated EV71 IRES-mediated translation with its helicase activity. Through an interaction with the truncated eIF4G, which binds to domain V, we proposed that DDX3X might be recruited to a region near domain VI and unwound the secondary structure of domain VI to facilitate ribosome entry. DDX3X also stimulated the translation mediated by the IRESs of coxsackievirus A16 (CA16), echovirus 9 (Echo 9), encephalomyocarditis (EMCV), and hepatitis C virus (HCV). Notably, EV71 infection significantly enhanced IRES-mediated translation, which was in part mediated by the protease activities of viral 2A^pro^ and 3C^pro^.

## Materials and Methods

### Lentivirus-Based shRNA Knockdown Screening

We searched for molecules important for EV71 replication by using a lentivirus-based shRNA library which targets whole human genome (provided by the RNAi Core, Taiwan). RD cells were first infected with pooled lentiviruses, followed by puromycin selection to select for lentivirus-transduced cells. The puromycin-resistant cells were then challenged with EV71 three rounds. Because EV71 infection causes cell lysis, the cells resistant to EV71-induced cell death might implicate a defective viral replication in the cells caused by shRNA knockdown, and the protein of the gene might represent the potential factor required for viral replication. Therefore, we harvested the survived cells when all of the control cells died from EV71 infection, extracted their genomic DNA, and PCR amplified the shRNA sequences from the genomic DNA using lentivirus vector-specific primers. We then cloned these PCR products into a bacterial plasmid, transformed them into bacteria, and performed DNA sequencing from individual bacterial clones. We have sequenced 88 clones in total and identified few candidate genes, including SCARB2 (26/88), CES2 (19/88), ACCSL (5/88), LIN7A (8/88), OR51B2 (2/88), DCLK3 (4/88), DDX3X (2/88), RNF5 (1/88), IRF3 (1/88), HSPA1 (1/88), and unknown (19/88). The numbers in the parenthesis indicate the frequencies of the gene found. We first excluded the genes which were not expressed in RD cells by RT-qPCR analysis (so they might be resulted from off-target effects), then validated the roles of the rest clones in EV71 replication cycle. DDX3X was chosen because it exhibited more stimulatory effects on EV71 replication than the others.

### Plasmids

The monocistronic reporter plasmid containing an EV71 IRES was modified from the pGL3-IRES-Luc plasmid (Huang et al., 2011) (kindly provided by Dr. Shin-Ru Shih, Chang-Gung University, Taiwan). Briefly, a firefly luciferase (Luc) cDNA containing a carboxy-terminal PEST (Pro, Glu, Ser, and Thr) sequence was used to replace the original Luc gene and a 3′-UTR of EV71 was added to the 3′ end of the Luc gene to generate the 5′UTR-LucPEST-3′UTR reporter DNA. The mutated IRESs (M1–M4) were constructed by a PCR site-directed mutagenesis method (Makarova et al., 2000) using the 5′UTR-LucPEST-3′UTR as a template, in which multiple CAA repeats (M1–M3) or mutated sequences (M4) were used to replace the original sequences in the mutated region. The CA16 and the Echo 9 IRES-Luc DNAs (Hou et al., 2016) and a dual reporter plasmid, which contained an EV71 IRES inserted between the *Renilla* and firefly luciferase DNAs, were obtained from Dr. Szu-Hao Kung (National Yang-Ming University, Taiwan). The EMCV IRES-Luc plasmid was derived from the pTM1 vector (Addgene) and the HCV IRES-Luc was described previously (Hwang et al., 1998).

The Flag-tagged DDX3X was constructed by cloning the cDNA of DDX3X into a CMV promoter-driven vector which contained a Flag tag, yielding the pcDNA3.1(+)/Flag-DDX3X-WT DNA. A shRNA-resistant DDX3X wobble DNA, which contained a 2-nucleotide mutation in the DDX3X shRNA (shDDX3X)-targeting region that did not alter the encoded amino acid, was constructed by PCR site-directed mutagenesis using pcDNA3.1(+)/Flag-DDX3X-WT as a template. The DQAD mutant, in which the DE_348_AD motif of DDX3X was mutated to DQ_348_AD, and the AAA mutant, in which the S_382_AT_384_ motif of DDX3X was mutated to A_382_AA_384_, were also constructed by PCR site-directed mutagenesis using the pcDNA3.1(+)/Flag-DDX3X-WT wobble DNA as a template.

To express the EV71 viral 2A, 2B, 2C, 3A, and 3D, the cDNA of each gene was PCR-amplified from an EV71 cDNA, SK-EV006 cDNA (Nishimura et al., 2009), and then cloned into the pEF-DEST51 vector (Addgene) which contributed a V5 tag to the carboxy-termini of the expressed proteins. Because the 2A protein cleaves eIF4G and cannot be expressed in a cap-dependent manner, we further subcloned the 2A-V5 DNA fragment into the pTM1 vector, yielding pTM1/2A-V5, in which the 2A expression was driven by the EMCV IRES. The protease-dead 2A and 3C mutants, which respectively had a C110A and a C147S mutations, were further constructed by PCR site-directed mutagenesis.

### Antibodies

The antibodies used in this study included rabbit anti-DDX3X (Catalog No. A300-474A-T) and anti-V5 antibodies from the Bethyl Company, mouse anti-DDX3X from Abcam (Catalog No. Ab196032), mouse anti-Flag and anti-β-actin antibodies from Sigma, mouse anti-eIF4G and control mouse IgG antibodies from BD, mouse anti-EV71 VP2 antibody from Millipore, and rabbit anti-G3BP antibody from Genetex. The anti-EV71-2C, 3C, and 3D mouse antisera were produced in our own laboratory using purified recombinant viral proteins.

### Cell Culture and Virus Infection

Human muscle rhabdomyosarcoma (RD), HEK293T, Huh7, and HeLa cells were maintained in Dulbecco’s modified Eagle’s medium (DMEM; Gibco) supplemented with 10% fetal bovine serum (FBS; Hyclone, GE Healthcare) at 37°C with 5% CO_2_. The EV71 strain (4643/TW/1998) was propagated in RD cells and infected cells at a multiplicity of infection (MOI) of 5. The viral titers were measured by plaque assays. DNA and RNA transfection in the cells were performed using lipofectamine 3000^TM^ (Invitrogen) following the manufacturer’s instructions. Lentiviruses expressing the control shRNA or the shRNA targeting DDX3X were purchased from the National RNAi Core Facility, Taiwan. To generate DDX3X knockdown cells, the cells were infected with shRNA-containing lentivirus at an MOI of 5 in the presence of polybrene (8 μg/ml). The infected cells were selected with puromycin (2.5 μg/ml) for 2 days and the resulting cells were used for subsequent experiments. All of the virus experiments were carried out in a bio-safety level 2 (BSL-2) laboratory, following the guidelines of the Center of Environmental Protection and Safety and Health, National Yang-Ming University, Taiwan.

### *In Vitro* Transcription

The IRES- or Cap-containing Luc reporter DNA or the pTM1/2A-V5 DNA was first linearized with the appropriate restriction enzymes. The resulting DNA fragments were *in vitro* transcribed using a MEGAscript^TM^ Kit or a mMESSAGE mMACHINE^TM^ Kit (which includes capping), and poly(A) tailed with a poly(A) Tailing Kit (for Cap-LucPEST RNA) (all from Invitrogen) following the manufacturer’s instructions to produce the IRES- or Cap- Luc reporter RNA or the EMCV IRES-directed 2A-V5 mRNA. To transcribe the RNA fragments from different regions of the IRES, we designed the forward and reverse PCR primers based on the sequences of the corresponding sites, and T7 promoter sequences were included at the 5′ end of each forward primer. The resulting PCR products were cleaned up and then directly used for *in vitro* transcription.

### Luciferase Activity Assays

The RD cells were mock-infected or infected with EV71 (MOI = 5) for 2 h followed by transfection with the *in vitro* transcribed 5′UTR-LucPEST-3′UTR or Cap-LucPEST reporter RNA. The cells were harvested at the indicated time points post viral infection. Half of the cells were lysed and the lysates were used for the luciferase activity assays using a Bright-Glo^TM^ Luciferase Assay System (Promega) according to the manufacturer’s instructions. One tenth of the cells were used for Alamar Blue assays to determine cell viability, and the rest of the cells were used for RNA extraction and the levels of the transfected reporter RNA were quantified by RT-qPCR to indicate transfectional efficiency. The luciferase activity was first normalized against the Alamar Blue activity, and then normalized against the reporter RNA levels to reflect the translational efficiency.

To analyze the effects of the EV71 viral proteins on the IRES activity, the RD cells were first transfected with the viral protein (2B, 2C, 3A, 3C, or 3D)-expressing plasmids. One day later, the cells were transfected with the *in vitro* transcribed 5′UTR-LucPEST-3′UTR RNA. The cell lysates were harvested at 6 h post RNA transfection and the luciferase activity was measured as described above. For the 2A protein effects, the RD cells were transfected with the 2A-V5 or the protease-dead 2A-V5 mRNA together with the IRES-Luc reporter RNA. The cell lysates were harvested for the luciferase activity assays at 6 h post RNA transfection. To analyze the effects of DDX3X on the other viral IRES activity, the individual IRES-containing Luc reporter RNAs were transcribed *in vitro* from the linearized DNAs, and the RNAs were subsequently transfected into cells with or without DDX3X knockdown (HeLa cells for CA16 IRES-Luc, Echo9 IRES-Luc, and EMCV IRES-Luc RNA, and Huh7 cells for HCV IRES-Luc RNA). Luciferase activity was assayed at 6 h post RNA transfection.

### Quantitative RT-PCR (RT-qPCR)

Total cellular RNA was extracted using TRIZOL^®^ reagent (Invitrogen) and cDNA was synthesized with HiScript I Reverse transcriptase (Bionovas Biotechnology Co. Ltd.). One-fifth of the volume of the cDNA product was subjected to qPCR with primers targeting to the 3D region of EV71 genome (forward: 5′- CCAAGATGAGCATGGAGGAT-3′; reverse: 5′- GATCTTGTCGATGGCCCTAA-3′), or to glyceraldehyde-3-phosphate dehydrogenase (*GAPDH*) (forward: 5′-GTATTGGGCGCCTGGTCACC-3′; reverse: 5′-CGCTCCTGGAAGATGGTGATGG-3′). qPCR was performed with Fast SYBR^®^ Green Master Mix (Thermo Fisher Scientific) according to the manufacturer’s instructions. The levels of viral RNA were normalized against those of *GAPDH*.

### *In Vitro* IRES Activity Assay and Antibody Blocking Experiments

The assays were conducted following a method previously reported (Hung et al., 2016). To prepare the translational extracts, the RD cells were grown to 90% confluence and then mock-infected or infected with EV71 (MOI = 5). At 6 h.p.i., the cells were harvested, and resuspended in 1.5x pellet volume of hypotonic lysis buffer which contained 10 mM HEPES-KOH (pH 7.6), 10 mM KOAc, 0.5 mM Mg(OAc)_2_, 2 mM DTT, and 1x protease inhibitor cocktail (Roche). The mixtures were placed on ice for 30 min and then homogenized with a 27-gauge 1/2-inch needle. Cell extracts were then centrifuged at 10,000 × *g* at 4°C for 20 min. The translational extracts were adjusted to a final concentration of 10 μg/μl using the lysis buffer and then stored at -80°C.

The *in vitro* IRES activity assays were performed in a final volume of 25 μl which contained 50 ng of 5′UTR-LucPEST-3′UTR RNA, 100 μg of translational extracts, 10 mM creatine phosphate, 50 μg/ml creatine phosphokinase, 79 mM KOAc, 0.5 mM Mg(OAc)_2_, 2 mM DTT, 0.02 mM hemin, 0.5 mM spermidine, 20 mM HEPES-KOH (pH 7.6), 20 μM amino acid mixture (Promega), 0.4 mM ATP, and RNase inhibitor. The reaction mixtures were incubated at 30°C for 90 min and the luciferase activity was measured by a Bright-Glo^TM^ Luciferase Assay System (Promega). For blocking experiments, the cell extracts were pre-incubated with control IgG or mouse anti-DDX3X antibody (Abcam) at the indicated doses at 4°C with mixing overnight before adding to the translational mixture.

### Protein Binding on CNBR-Activated RNA Affinity Beads

The binding assays were conducted following a previously described method (Vashist et al., 2012). Briefly, 30 mg of cyanogen bromide (CNBR)-activated Sepharose beads (Sigma) were pre-swollen in 1 ml of cold 1 mM HCl for 1 h. After centrifugation at 500 × *g* for 30 s, the beads were washed three times with 1 ml of cold 200 mM 4-morpholineethanesulfonic acid (MES), pH 6.0. The packed pre-swollen CNBR-activated beads (25 μl) were resuspended in 100 μl of 200 mM MES buffer and mixed with 20 μg of the *in vitro* transcribed RNA. The mixture was kept at 4°C with mixing for 14–16 h. The beads were washed three times with cold MES buffer and then the unreacted groups were blocked with 1 ml of 0.2 M glycine at room temperature for 2 h. The RNA-conjugated beads were washed three times with 1 ml of cold binding buffer (50 mM HEPES, pH 7.6, 20 mM KCl, 5 mM MgO-acetate, 50 mM NaCl, 2 mM DTT, and 10% glycerol) and ready for lysate binding. The cells were lysed in CHAPS lysis buffer (10 mM Tris-HCl, pH 7.4, 1 mM MgCl_2_, 1 mM EGTA, 0.5% CHAPS, 10% glycerol, 1 mM DTT, and 1x protease inhibitor). RNaseOUT^TM^ (20 U) was first added to 500 μg of cell lysates. The lysates were then mixed with the RNA-coupled CNBR-activated beads, and the mixture was incubated at 4°C for 2 h with mixing. The protein-RNA complexes bound to the beads were washed three times with cold binding buffer, and the bound proteins were eluted by boiling in Laemmli sample buffer. The proteins were resolved on a 12.5% SDS-PAGE gel, followed by western blot analysis.

### Co-immunoprecipitation

To analyze protein-protein interactions on EV71 IRES RNA, proteins were first bound to the CNBR-activated RNA beads as described above. The RNA-protein complexes bound on the Sepharose beads were washed three times with binding buffer and the proteins were eluted by an RNase A treatment at room temperature for 4 h. The eluted proteins were resuspended in 750 μl of immunoprecipitation (IP) buffer (50 mM Tris-HCl, pH 7.4, 100 mM NaCl, 0.2% Nonidet P-40, 10 mM NaF, and 1 mM EDTA) and 2 μg of mouse anti-DDX3X antibody (Abcam) or a control IgG antibody was added. The mixtures were incubated at 4°C overnight. Subsequently, 10–20 μl of protein-G Sepharose beads (GE Healthcare) were added, and the samples were incubated at 4°C for another 2 h. The beads were washed three times with ice-cold IP buffer. The protein samples were heated at 95°C for 5 min in Laemmli sample buffer and then subjected to western blot analysis.

### Statistical Analysis

The data were analyzed by a two-tailed Student’s *t*-test or one-way ANOVA (Graphpad Prism 5 Software). ^∗∗∗^ indicates *P <*0.001, ^∗∗^ indicates *P <*0.01, and ^∗^ indicates *P <*0.05. *P <*0.05 was regarded as statistically significant.

## Results

### EV71 Infection Significantly Up-Regulates IRES-Dependent Translation and DDX3X Knockdown Reduces the Translation

**FIGURE 1:**
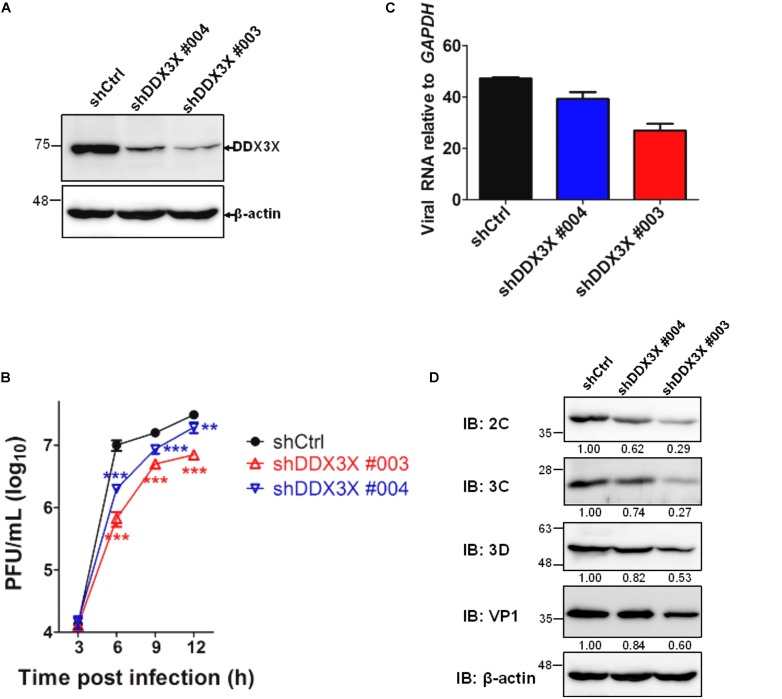
DDX3X is a positive regulator of EV71 replication. RD cells were first infected with lentiviruses expressing control shRNA (shCtrl) or shRNAs targeting DDX3X (shDDX3X#003 and shDDX3X#004). After 2 days of selection with puromycin, the cells were infected with EV71 (MOI = 5). **(A)** The knockdown efficiency of shDDX3X#003 and shDDX3X#004 analyzed by western blot analysis is shown. **(B)** EV71 viral titers were measured by plaque assays from the supernatant and cell lysates of EV71-infected cells at the indicated time points post-viral infection. **(C)** Viral RNA levels in EV71-infected cells were measured by RT-qPCR at 6 h.p.i. **(D)** Expression of the viral proteins in EV71-infected cells was analyzed at 6 h.p.i. using mouse antibodies against 2C, 3C, 3D, and VP1. The relative expression levels are indicated by the numbers. The western blot analysis of β-actin, used as a loading control, was shared in **(A,D)**. In **(B,C)**, one-way ANOVA followed by Dunnett’s *post hoc* analysis was performed for comparison between the shCtrl group and the DDX3X knockdown groups. ^∗^*P <*0.05, ^∗∗^*P <*0.01, ^∗∗∗^*P <*0.001.

We have employed a lentivirus-mediated shRNA knockdown strategy to screen for novel molecules involved in the EV71 replication cycle (see Materials and Methods). DDX3X was identified as an important molecule because its knockdown rendered RD cells resistant to EV71-induced cell lysis. We further investigated the role of DDX3X in the EV71 replication cycle. The results shown in **Figure 1** indicated that lentivirus-mediated DDX3X silencing by two independent shRNA clones (shDDX3X#004 and shDDX3X#003, **Figure 1A**) significantly reduced the EV71 viral titers (**Figure 1B**), viral RNA levels (**Figure 1C**), and viral protein expression (**Figure 1D**), which was especially evident at 6 h post infection (h.p.i.). Notably, the reductions of viral titer, viral RNA and protein levels all correlated with the decrease in DDX3X protein levels. These results suggest that DDX3X may act as a positive regulator of EV71 replication.

**FIGURE 2:**
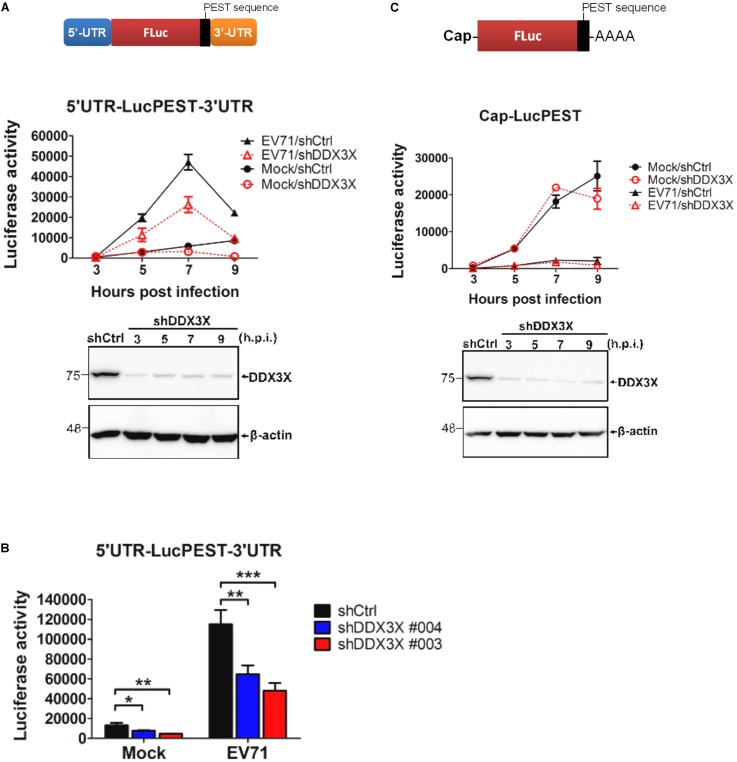
EV71 infection stimulates EV71 IRES-dependent translation and DDX3X facilitates the translation. A schematic of 5′UTR-LucPEST-3′UTR monocistronic reporter RNA **(A)**, or Cap-LucPEST reporter RNA **(C)**, is shown on the top. The RD cells, treated with shCtrl or shDDX3X#003 **(A,C)**, or with shCtrl, shDDX#003 or shDDX3X#004 **(B)**, were mock-infected or infected with EV71 at an MOI of 5. After viral infection, the cells were further transfected with the *in vitro* transcribed 5′UTR-LucPEST-3′UTR reporter RNA **(A,B)** or Cap-LucPEST reporter RNA **(C)**. The cells were harvested at the indicated time points post-EV71 infection **(A,C)** or at 7 h.p.i. **(B)**. Half of the cells were used for luciferase activity assays, 1/10 for Alamar Blue assay to determine cell viability, and the rest were used for RNA quantification by RT-qPCR to determine the RNA levels. The luciferase activity was normalized against Alamar Blue value and the RNA levels. All the results presented are the mean ± SD of three independent experiments. One-way ANOVA followed by Bonferroni’s correction was performed. In **(A)**, the statistical analyses indicated ^∗∗∗^*P <*0.001 for EV71/shCtrl vs. Mock/shCtrl at all time points except 3 h.p.i., ^∗∗∗^*P <*0.001 for EV71/shCtrl vs. EV71/shDDX3X at 7 h.p.i., and ^∗∗∗^*P <*0.001 for Mock/shCtrl vs. Mock/shDDX3X at 9 h.p.i.

To investigate whether the DDX3X positive regulation of EV71 replication is at the mRNA translational level, we used a monocistronic reporter RNA, 5′UTR-LucPEST-3′UTR in which the translation of luciferase was driven by the EV71 5′-UTR (**Figure 2A**), to monitor viral IRES activity. The firefly luciferase protein expressed from the construct harbors a PEST (proline, glutamic acid, serine, and threonine) peptide sequence at the carboxyl-end, which gives the expressed protein a short half-life and allows the measured luciferase activity to truly reflect the translational activity rather than the accumulated luciferase protein level. Translation was conducted in the presence or absence of EV71 infection. The RD cells treated with control shRNA (shCtrl) or shRNA targeting DDX3X (shDDX3X) were mock-infected or infected with EV71 (MOI = 5), followed by transfection with the *in vitro* transcribed 5′UTR-LucPEST-3′UTR reporter RNA. The results shown in **Figure 2A** revealed that EV71 IRES activity was markedly enhanced by an EV71 infection in control knockdown or DDX3X knockdown RD cells, evident at 7 h.p.i. (*p* < 0.001, EV71 vs. mock). Notably, DDX3X knockdown significantly reduced the IRES activity in EV71-infected cells (*p* < 0.001, EV71/shCtrl *vs*. EV71/shDDX3X at 7 h.p.i.), and also in mock-infected cells (*p* < 0.001, Mock/shCtrl *vs*. Mock/shDDX3X at 9 h.p.i.) albeit the IRES activity was low in the latter. The IRES activity, in the presence or absence of EV71 infection, also decreased with the decrease of DDX3X in a dose-dependent manner (**Figure 2B**).

It was noted that the effects of DDX3X knockdown on viral titers (**Figure 1B**) or IRES-Luc activity (**Figure 2A**) were transient. Our explanation is that, unlike the canonical translational initiation factors, DDX3X is not “essential” to, but just stimulates, the translation. Thus, DDX3X silencing only reduced IRES translation to some extents (i.e., ∼50% shown here) and did not abolish it completely. Given our infection condition (MOI = 5) with a fixed number of cells, viral titers reached to a plateau by 6–12 h.p.i. in control knockdown cells (**Figure 1B**) and cells started to die around 9–12 h.p.i. Notably, viral titer rose more slowly in DDX3X knockdown cells but also could reach to a plateau around 9–12 h.p.i. Since control knockdown cells stopped producing new viral particles earlier than DDX3X knockdown cells, the viral titer in the latter eventually approached to that of the control knockdown cells at the final time points, especially in the cells treated with the less effective shDDX3X#004. As for the transient luciferase activity, it is possibly due to the transient stability, and thus the translation, of the transfected reporter RNA in the cells.

A Cap-LucPEST RNA without EV71 IRES was *in vitro* transcribed, capped, and tailed with poly(A) (**Figure 2C**), and conducted similar experiments as described above to analyze the effect of DDX3X knockdown on cap-dependent translation. As anticipated, an EV71 infection dramatically suppressed the cap-dependent translation (**Figure 2C**, *p* < 0.001, Mock vs. EV71). In the mock-infected cells, DDX3X silencing seemed to have little impact on the cap-dependent translation up to 7 h.p.i. Though there were greater variations on the Luc values at 9 h.p.i., the difference still did not reach a statistical significance. Thus, we tempted to believe that DDX3X had no impacts on the unstructured cap-dependent translation, which was in agreement with most of previous studies (Lai et al., 2008, 2010; Soto-Rifo et al., 2012), proposing that DDX3X was not involved in general cap-dependent translation. Even if DDX3X might have some impacts on the Cap-Luc activity shown in **Figure 2C**, we speculated that the results might be caused by a secondary effect, as the difference appeared quite late. However, it is unclear currently what this secondary effect may be. Together, all these data demonstrate that the EV71 IRES activity is significantly stimulated by viral infection and that DDX3X is important for the IRES-dependent translation but not the cap-dependent translation.

### The Helicase Activity of DDX3X Is Crucial for Its Stimulatory Effects on EV71 IRES-Mediated Translation

**FIGURE 3:**
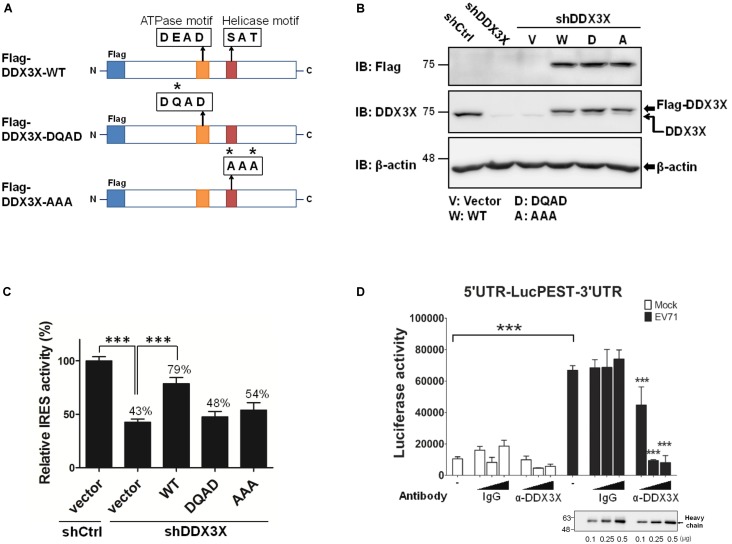
DDX3X employs its helicase activity to stimulate IRES-dependent translation. **(A)** A schematic of DDX3X helicase mutants is shown. Asterisks indicate the sites that were mutated. The RD cells depleted of DDX3X (shDDX3X) or not (shCtrl) were mock transfected or transfected with the vector, or shDDX3X-resistant Flag-DDX3X-WT (W), Flag-DDX3X-DQAD (D), or Flag-DDX3X-AAA (A) plasmids together with a dual reporter plasmid containing EV71 IRES. **(B)** A western blot analysis indicates the remaining levels of endogenous DDX3X after DDX3X knockdown detected by an anti-DDX3X antibody (the lower arrow in the second panel). The levels of ectopically expressed Flag-DDX3X WT or Flag-DDX3X mutants were detected by an anti-Flag antibody or an anti-DDX3X antibody (the upper arrow in the second panel). **(C)** The luciferase activity was measured at 24 h post DNA transfection. The Luc value in the shCtrl cells transfected with vector DNA was arbitrarily set as 100%. The luciferase activities of all the other groups were normalized against the shCtrl/vector group and are expressed as relative IRES activity (%). The results expressed are the mean ± SD of three independent experiments. ^∗^*^∗∗^P <*0.001 between the indicated groups (One-way ANOVA followed by Dunnett’s *post hoc* test). **(D)** The translational extracts prepared from mock-infected or EV71-infected cells were pre-incubated with 0, 0.1, 0.25, or 0.5 μg of control mouse IgG or mouse anti-DDX3X antibody, followed by incubation with the *in vitro* transcribed 5′UTR-LucPEST-3′UTR RNA at 30°C for 90 min. The *in vitro* translational products were then subjected to luciferase activity assays. The results expressed are the mean ± SD of three independent experiments. ^∗^*^∗∗^P <*0.001 between the indicated groups and the equivalent groups treated with the same doses of control IgG in the lysates of EV71-infected cells (One-way ANOVA followed by Bonferroni’s correction).

To understand whether the RNA helicase activity of DDX3X was important for its stimulatory effect on the IRES-dependent translation, we employed two DDX3X mutants, one mutant lacked ATPase activity (the DQAD mutant) and the other lacked RNA helicase activity (the AAA mutant) (**Figure 3A**) (Shih et al., 2008). The endogenous DDX3X was first depleted from RD cells by shDDX3X-expressing lentivirus; the cells were then reconstituted with a control vector or shDDX3X-resistant Flag-DDX3X-WT, Flag-DDX3X-DQAD, or Flag-DDX3X-AAA. As shown in **Figure 3B**, the levels of endogenous DDX3X were significantly reduced by shDDX3X (indicated by the lower arrow), whereas the expression levels of the transfected wild-type or DDX3X mutants were more or less comparable (the upper arrow). The results of the luciferase activity analysis revealed that reconstitution of the DDX3X knockdown cells with Flag-DDX3X-WT protein, but not with the Flag-DDX3X-DQAD or Flag-DDX3X-AAA mutant protein, significantly restored the IRES activity (**Figure 3C**). These results demonstrate that the helicase activity of DDX3X is important for its stimulatory effects on the IRES activity.

To exclude the possibility that the above findings were resulted from a secondary effect of DDX3X silencing *in vivo*, we further performed *in vitro* translation assays using the 5′UTR-LucPEST-3′UTR reporter RNA in the presence or absence of DDX3X-specific antibody. The translational extracts were prepared from mock-infected or EV71-infected cells (Hung et al., 2016). Analogous to the *in vivo* results, the extracts of EV71-infected cells exhibited much higher translational activity than those of mock-infected cells (*P* < 0.001, **Figure 3D**), which implicated the presence of a more efficient translational complex in the former. Moreover, the anti-DDX3X antibody, but not control IgG, markedly blocked the IRES-mediated translation in the extracts of EV71-infected cells, suggesting a direct role of DDX3X in the IRES-mediated translation.

### Specific Binding of DDX3X to IRES RNA May Be Mediated by Interacting With eIF4G

**FIGURE 4:**
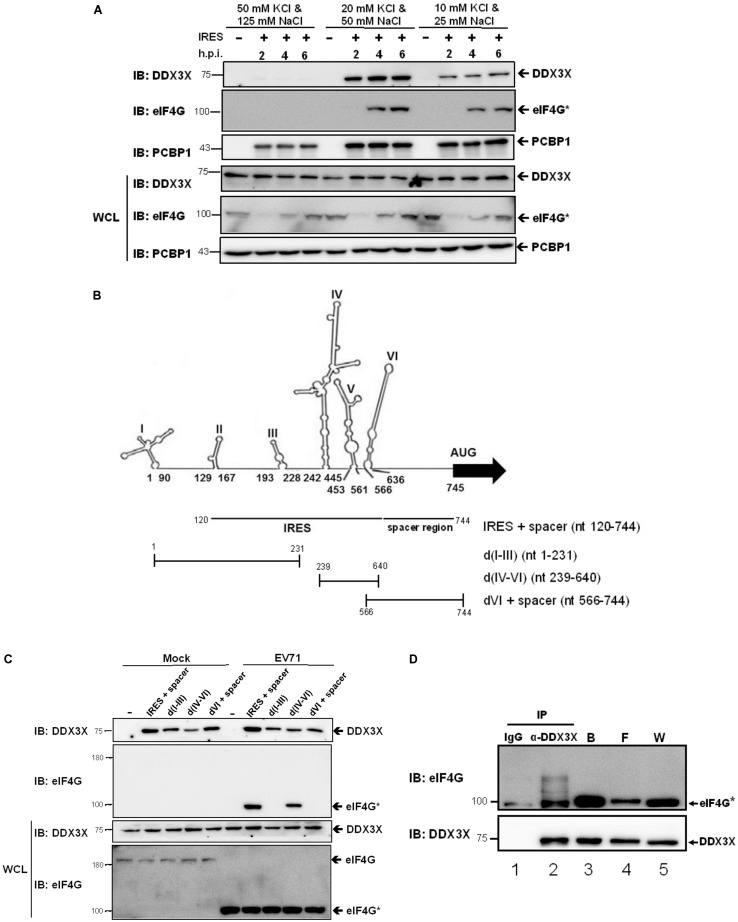
DDX3X interacts with the truncated eIF4G on the IRES RNA. **(A)** The *in vitro* transcribed RNA fragment, containing IRES and a spacer region, was coupled to the CNBR-activated Sepharose beads as described in the Materials and Methods. The cell lysates, harvested at 2, 4, and 6 h.p.i., were incubated with the RNA-coupled Sepharose beads under the indicated salt conditions and the proteins bound on the beads were analyzed by western blot using anti-DDX3X, anti-eIF4G, or anti-PCBP1 antibody. The cleaved eIF4G was shown as eIF4G^∗^. WCL, whole cell lysate. **(B)** Schematic representations of the RNA fragments derived from the different portions of EV71 5′-UTR are shown. The IRES + spacer encompasses nt 120–744. The d(I-III) encompass nt 1–231, d(IV-VI) encompass nt 239–640, and dVI + spacer encompass nt 566–744. **(C)** DDX3X can bind to all parts of the 5′-UTR RNA *in vitro*. The RNA fragments described in **(B)** were coupled to the CNBR-activated Sepharose beads. EV71-infected or mock-infected cell lysates were incubated with the RNA-conjugated beads under the intermediate salt condition (20 mM KCl and 50 mM NaCl) and the proteins bound were analyzed by western blot using anti-DDX3X or anti- eIF4G antibody. **(D)** DDX3X interacts with the truncated eIF4G on the IRES RNA. The IRES RNA fragment (nt 120–744) conjugated on the CNBR-activated beads was used to pull down the proteins bound to the RNA, which were then eluted with RNase A. A co-immunoprecipitation experiment was conducted on the eluted proteins using control IgG or anti-DDX3X antibody. The immunoprecipitates were analyzed by western blot using anti-eIF4G and anti-DDX3X antibodies. W, whole cell lysate; F, flow through; B, the proteins that bound on the RNA-conjugated Sepharose beads.

Since DDX3X employed its helicase activity to stimulate IRES activity, we expected it to have an RNA binding activity. To prove this thought and to understand whether DDX3X has binding specificity, we performed *in vitro* RNA binding assays. The RNA fragment (nt 120-744), encompassing the IRES (domains II–VI) and the spacer regions, was *in vitro* transcribed and subsequently coupled to cyanogen bromide (CNBR)-activated Sepharose beads (Vashist et al., 2012). Cell lysates of EV71-infected RD cells were incubated with the RNA-coupled Sepharose beads under different salt conditions. After an extensive wash, the cellular proteins bound to the beads were eluted and resolved by SDS-PAGE. DDX3X was detected by a western blot analysis. It was found that DDX3X bound to IRES RNA inefficiently under high salt conditions (50 mM KCl + 125 mM NaCl) but efficiently under intermediate (20 mM KCl + 50 mM NaCl) or low salt conditions (10 mM KCl + 25 mM NaCl) (**Figure 4A**). Interestingly, the truncated eIF4G, generated when an EV71 infection was present, also bound very inefficiently to IRES RNA under high salt conditions, whereas PCBP1, a previously reported ITAF (Choi et al., 2004), bound efficiently under all salt conditions. The results suggest that unlike the ITAFs, DDX3X and eIF4G only have a moderate affinity for RNA binding.

Next, we examined whether DDX3X bound to a specific region of the IRES. We performed RNA binding assays using the RNA fragments derived from the different portions of the EV71 5′-UTR, including the IRES + spacer, domain (d) I-III, d(IV-VI), and dVI + spacer (**Figure 4B**). The results showed that DDX3X bound efficiently to all of these RNA fragments irrespective of an EV71 infection (**Figure 4C**). On the other hand, the truncated eIF4G bound specifically to d(IV-VI), which was in agreement with previous finding that it bound to domain V of the enteroviral IRES (de Breyne et al., 2009). Taken together, the results demonstrate that DDX3X has no RNA binding preference *in vitro*.

Given that DDX3X has only a moderate affinity for RNA binding, we envisaged that it may need an interacting partner(s) to be recruited to a specific region of the IRES for the *in vivo* RNA unwinding. The truncated eIF4G was first considered since it has been reported that DDX3X interacts with eIF4G and cooperates with eIF4A to destabilize the TAR region of HIV genomic RNA, allowing the attachment of the 43S ribosome complex to the 5’-end of HIV mRNA (Soto-Rifo et al., 2012). Thus, we performed co-immunoprecipitation experiments. The IRES RNA fragment was used to first pull down the IRES RNA-binding proteins. As expected, the truncated eIF4G and DDX3X were found in the bound fraction (**Figure 4D**, lane 3). The proteins bound to the IRES RNA were then eluted by RNase A treatment, which also degraded all of the RNA, followed by co-immunoprecipitation using control IgG or anti-DDX3X antibody. We found that the truncated eIF4G was significantly co-immunoprecipitated by the anti-DDX3X antibody (lane 2), albeit it also had slight non-specific interaction with control IgG (lane 1). These results indicated that the truncated eIF4G could associate with DDX3X independent of the RNA. Taken together, we predicted that DDX3X might bind to the IRES *in vivo via* an interaction with the truncated eIF4G.

### DDX3X May Unwind Domain VI to Promote EV71 IRES Activity

**FIGURE 5:**
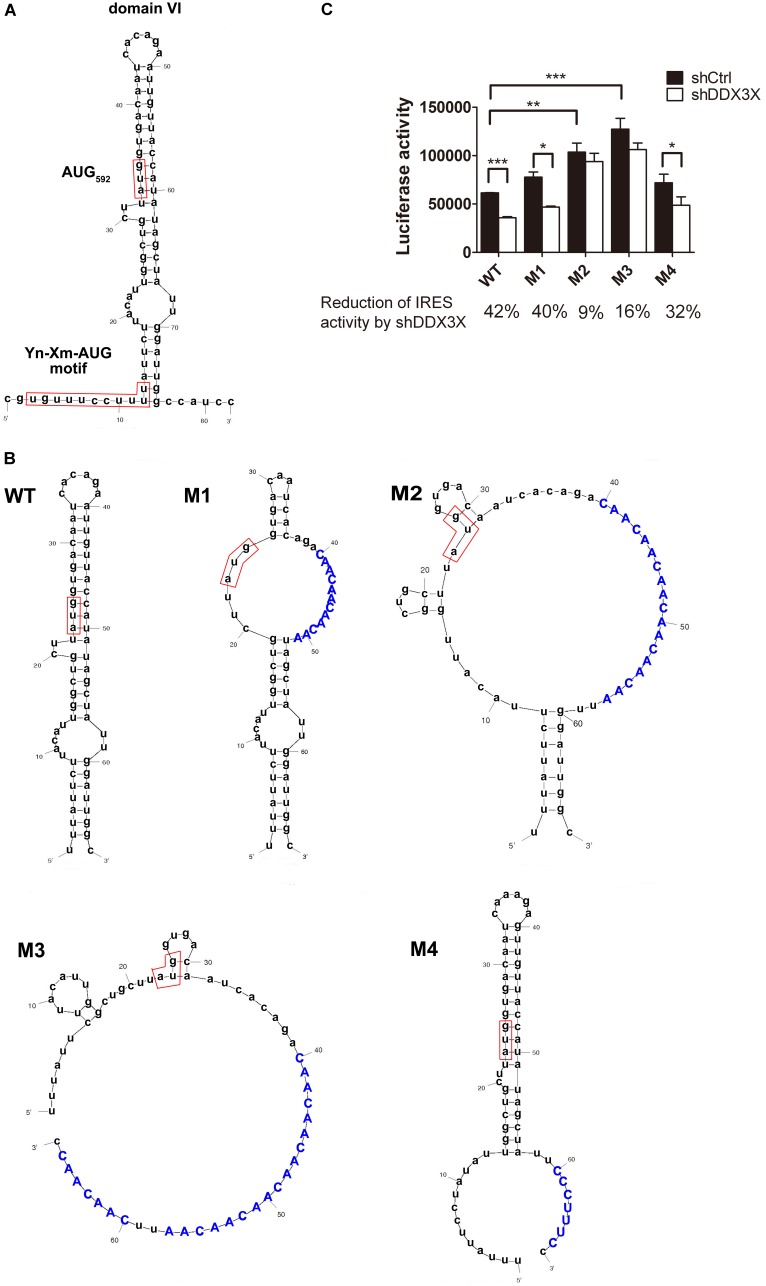
DDX3X unwinds domain VI of EV71 IRES to facilitate IRES-dependent translation. **(A)** The secondary structure of domain VI of EV71 IRES is shown. The conserved Yn-Xm-AUG_592_ motif is highlighted in the boxes. **(B)** The secondary structures of domain VI for the wild-type (WT) and mutants M1–M4 predicted by M-fold software are shown. The mutated sequences are shown in capital letters. **(C)** DDX3X dependences of various IRES mutants were determined. The RD cells depleted of DDX3X (shDDX3X) or not (shCtrl) were infected with EV71 (MOI = 5); the cells were then transfected with the *in vitro* transcribed WT or mutant IRES-Luc reporter RNAs and the luciferase activity was measured at 6 h post EV71 infection. The reduction percentage of the luciferase activity by DDX3X knockdown for each mutant IRES is shown at the bottom. The results expressed are the mean ± SD of three independent experiments. Two-tailed Student’s *t*-test was performed for the comparison between the shCtrl and the shDDX3X groups within each IRES mutant, whereas one-way ANOVA followed by Dunnett’s *post hoc* test was performed between the indicated groups. *^∗^P <*0.05, *^∗∗^P <*0.01, ^∗^*^∗∗^P <*0.001.

The secondary structures of most of the EV71 IRES domains are thought to be important for IRES activity (Thompson and Sarnow, 2003), and the conformations are maintained by many ITAFs. On the other hand, the secondary structure of domain VI, where the hypothesized ribosome landing pad resides (**Figure 5A**), may actually hinder the accessibility of AUG_592_ to the ribosome and reduce the efficiency of the formation of the 48S complex (Sweeney et al., 2014). Since the truncated eIF4G binds specifically to domain V, we speculated that DDX3X might be recruited to domain VI or a region nearby *via* an interaction with eIF4G and locally unwind the secondary structure of domain VI, thus facilitating ribosome entry and scanning. If this is the case, we would predict that the destabilization of domain VI might lead to increased IRES activity, and the IRES-dependent translation might become DDX3X independent.

To test this hypothesis, we constructed four mutant IRESs, M1-M4 (**Figure 5B**) and used them to drive luciferase gene expression. We chose to use multiple CAA repeats to mutate the sequences on the opposite side of the AUG-containing stem of domain VI to avoid the destruction of the conserved Yn-Xm-AUG motif, while the mutation in these four mutant IRESs might cause different degrees of impairment in their secondary structures, according to the M-fold software prediction (**Figure 5B**). The luciferase activities directed by these mutant IRESs were determined in control knockdown or DDX3X knockdown RD cells. The results showed that DDX3X knockdown reduced the translational activities of IRESs M1 and M4 by 40 and 32%, respectively (**Figure 5C**, *p* < 0.05, shCtrl vs. shDDX3X), in a similar manner to that of the WT IRES (42%), but it only reduced the translational activities of M2 and M3 by 9 and 16%, respectively, which did not reach a statistical significance (**Figure 5C**). The results implicated that DDX3X was no longer important for the translation directed by IRESs M2 and M3. It is noteworthy that the translational activities of M2 and M3 were significantly increased compared to WT IRES, even in the control knockdown cells. These results strongly suggest that the secondary structure of domain VI hinders the IRES-mediated translation and that DDX3X may utilize its helicase activity to unwind the secondary structure.

### The 2A^pro^ and 3C^pro^ of EV71 Enhance IRES-Mediated Translation

**FIGURE 6:**
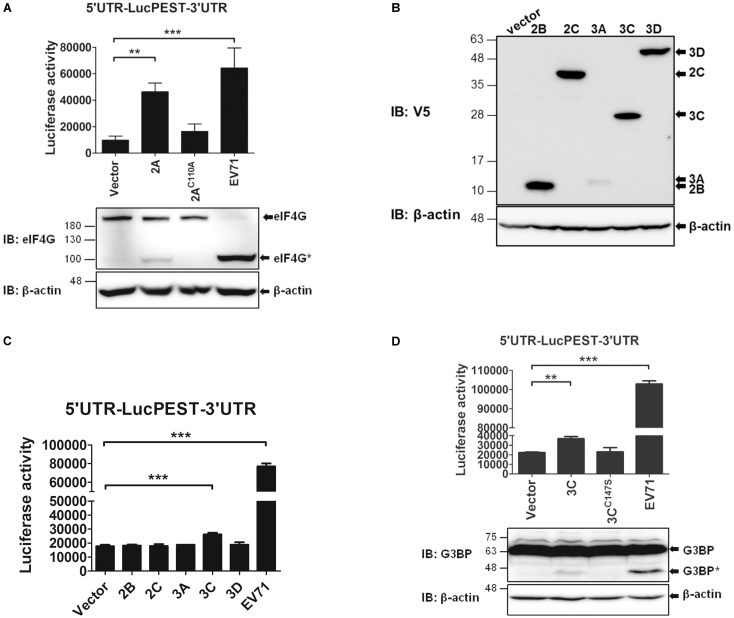
The 2A^pro^ and 3C^pro^ of EV71 stimulate IRES-mediated translation. **(A)** 2A^pro^ employs its protease activity to enhance the IRES activity. RD cells were co-transfected with the *in vitro* transcribed 5′UTR-LucPEST-3′UTR reporter RNA and 2A RNA or the protease-dead (2A^C110A^) RNA. Cells infected with EV71 (MOI = 5) and transfected with 5′UTR-LucPEST-3′UTR reporter RNA served as positive controls. The luciferase activity was measured at 6 h post RNA transfection or EV71 infection. In a separate experimental set, the cells were harvested at 6 h post RNA transfection or EV71 infection and the protein lysates were analyzed by western blot using anti-eIF4G antibody. The cleaved eIF4G shown as eIF4G^∗^ indicated the 2A^pro^ protease activity. **(B)** Expression of the EV71 viral proteins. The cDNAs of EV71 2B, 2C, 3A, 3C, and 3D cloned in pEF-DEST51 vector, which contributed a V5 Tag to the C-termini of the expressed proteins, were expressed in the RD cells. The cell lysates harvested at 24 h after DNA transfection were analyzed by western blot using anti-V5 antibody. **(C,D)** 3C^pro^ employs its protease activity to enhance the IRES activity. RD cells were first transfected with the 2B-, 2C-, 3A-, 3C-, and 3D-expressing plasmids **(C)** or with 3C- and the protease dead 3C^C147S^-expressing plasmids **(D)**. Twenty four hours later, the cells were transfected with the 5′UTR-LucPEST-3′UTR reporter RNA. Cells infected with EV71 and transfected with 5′UTR-LucPEST-3′UTR reporter RNA served as positive controls. In a separate experimental set, cells were harvested at 6 h post RNA transfection or EV71 infection, and protein lysates were analyzed by western blot using antibody against G3BP. The cleaved G3BP shown as G3BP^∗^ indicated the 3C^pro^ protease activity. All the results presented are the mean ± SD of three independent experiments. ^∗^*p* < 0.05, *^∗∗^P <*0.01, ^∗^*^∗∗^P <*0.001 between the indicated groups (One-way ANOVA followed by Dunnett’s *post hoc* test).

The results shown in **Figure 2A** indicate that an EV71 infection markedly enhances IRES activity. Therefore, we wanted to investigate which viral proteins might contribute to this enhancement. First, we examined the effects of EV71 2A^pro^ on the IRES activity. Because 2A^pro^ cleaves eIF4G and shuts off cap-dependent translation, we expressed 2A^pro^ in the pTM1 vector, in which the expression of 2A^pro^ was driven by the EMCV IRES. The *in vitro* transcribed EMCV IRES-2A^pro^ RNA or the protease-dead mutant (EMCV IRES-2A^C110A^) RNA was co-transfected with the 5′UTR-LucPEST-3′UTR reporter RNA into the RD cells. However, due to the low levels of 2A expressed from the transfected RNA, preventing us from directly detecting the protein by using an antibody against V5, a tag at the C-terminus of the expressed protein, we employed an activity assay instead to indicate the expression of 2A^pro^ protein. 2A^pro^ activity was revealed by the cleavage of eIF4G protein examined by a western blot analysis (**Figure 6A**). As expected, the cleaved eIF4G was detected in the cells expressing the wild-type 2A^pro^ but not the 2A^C110A^ mutant, albeit the levels were much lower than in the EV71-infected cells in which viral RNA could replicate continuously and produced much higher levels of 2A^pro^ proteins. The data showed that 2A^pro^ expression significantly enhanced EV71 IRES activity and that the protease activity was required for the enhancement (**Figure 6A**). Next, we examined the effects of the other viral proteins on the EV71 IRES activity. The cDNAs of EV71 2B, 2C, 3A, 3C, and 3D were cloned in the pEF-DEST51 vector, which contributed a V5 Tag to the C-termini of the proteins. The results of a western blot analysis using the anti-V5 antibody revealed that all proteins but the 3A protein were expressed efficiently in the cells (**Figure 6B**). Under the conditions that each viral protein was expressed in isolation, the IRES-luciferase activity was slightly enhanced by 3C^pro^ but not by the other viral proteins (**Figure 6C**); however, the effect of 3A remains to be elucidated. Again, the protease activity of 3C^pro^ was required for the enhancement (**Figure 6D**). In a similar way, the activity of 3C^pro^ was revealed by the cleavage of Ras-GAP SH3 domain-binding protein (G3BP), a substrate of EV71 3C^pro^ (White et al., 2007). Taken together, these results indicate that both viral 2A^pro^ and 3C^pro^ enhance IRES-mediated translation *via* their protease activities, which probably cleave and/or generate some cellular factors to stimulate the translation (see Discussion).

### DDX3X Also Facilitates the Translation of Other Types of IRES

**FIGURE 7:**
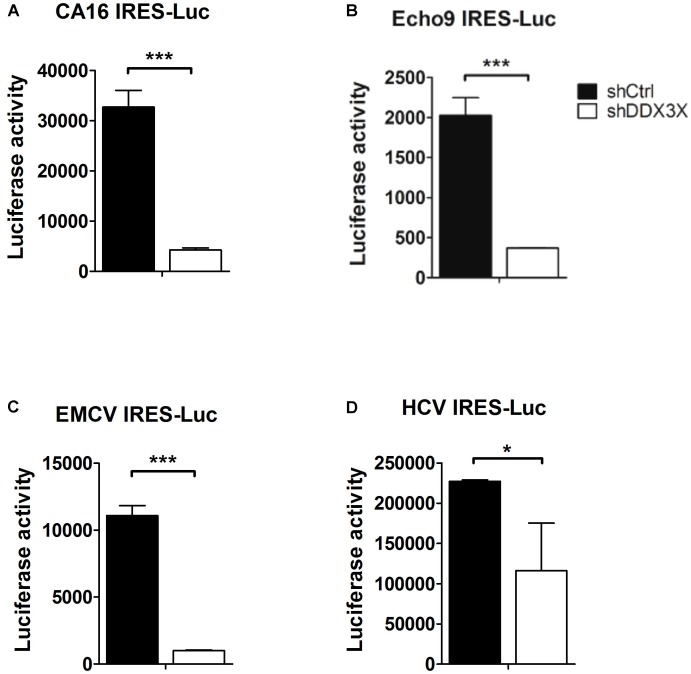
DDX3X stimulates the translation of other types of viral IRESs. The IRESs that direct the translation of luciferase reporter in the monocistronic constructs were derived from **(A)** CA16 virus, **(B)** Echovirus 9, **(C)** EMCV, and **(D)** HCV. The former three IRES-Luc RNAs were transfected into HeLa cells, whereas HCV IRES-Luc RNA was transfected into Huh7 cells. Both HeLa and Huh7 cells were pre-treated with lentiviruses expressing shCtrl or shDDX3X. The luciferase activity was measured at 6 h post RNA transfection. Results presented are the mean ± SD of three independent experiments. *^∗^P <*0.05, ^∗^*^∗∗^P <*0.001 between the shCtrl and the shDDX3X groups (Student’s *t*-test).

It was reported that DDX3X interacts with several translational initiation factors, such as eIF4E (Shih et al., 2008), eIF4G, eIF2α, PABP (Lai et al., 2008), eIF3 (Lee et al., 2008; Geissler et al., 2012), and a small ribosomal protein rpS6 (Geissler et al., 2012). So, we wanted to investigate whether DDX3X also could stimulate the translation of other types of IRES because most IRESs also contain complex structures near the AUG initiation site and their activities depend on some canonical translational factors as well. We chose IRESs from coxsackievirus A16, Echovirus 9, EMCV, and HCV. The luciferase activities driven by these viral IRESs were determined in control knockdown or DDX3X knockdown cells. The results demonstrated that depletion of DDX3X also significantly impacted the IRES activities of CA16, Echovirus 9, and EMCV, and moderately affected the IRES activity of HCV (**Figure 7**). Therefore, DDX3X seems to be generally required for the translation of these highly structured viral IRESs.

## Discussion

The domain VI of PV or EV71 IRESs harbors the conserved Yn-Xm-AUG motif, which has been hypothesized to be the ribosome landing pad (Nicholson et al., 1991; Pilipenko et al., 1992). Previous studies have demonstrated that mutations in the motif sequences significantly impair the IRES activity, which cannot be rescued by compensatory mutations that restore the secondary structure (Meerovitch et al., 1991; Nicholson et al., 1991). These results indicate the importance of these motif sequences in regulating the IRES activity. However, the stem-loop structure of domain VI may actually hinder the accessibility of the AUG_592_ triplet to ribosome and thus reduces viral mRNA translation. It has been established that the binding of eIF4G/eIF4A to domain V induces conformational changes downstream of the binding site, which stimulates the recruitment of the ribosome complex (de Breyne et al., 2009). Normally, upon cap structure recognition, eIF4A is thought to be responsible for the unwinding of the secondary structure to allow ribosome movement. However, several cellular mRNAs that have a secondary structure at their 5’ ends are resistant to the unwinding activity of eIF4A (Kozak, 1991; Pickering and Willis, 2005) and require some additional helicase, such as DHX29 or DHX9, to help eIF4A unwind the secondary structure to enhance ribosome scanning (Parsyan et al., 2011). Considering that the domain VI of EV71 IRES contains a long stem-loop structure and the AUG triplet is sequestered in it, it is anticipated that it may also need an additional helicase to facilitate 43S ribosome landing and movement on EV71 IRES. In *in vitro* reconstitution experiments, Sweeney et al. (2014) have ruled out the possibility that DHX29 participates in the unwinding of domain VI of EV71 IRES, leaving the helicase candidate undiscovered.

In this study, we provide evidence demonstrating that DDX3X may be a potential candidate for this role. We chose to mutate the sequences opposite the Yn-Xm-AUG motif side to disrupt the stem structure of domain VI. Interestingly, when the stem-loop structure was largely disrupted, DDX3X was no longer important for the IRES activity. In particular, the IRES translational activity proportionally increased with the degree of impairment of the secondary structure of domain VI (**Figure 5**). Thus, we propose that DDX3X functions to destabilize the secondary structure of domain VI, so that the AUG_592_ triplet sequestered in it becomes accessible to the 43S ribosome complex, and the IRES-dependent translation can be stimulated. We also demonstrated that DDX3X enhanced the translational activity of CA16, Echo 9, EMCV, and HCV IRESs with different efficiencies. Analogous to EV71 IRES, the ribosome entry sites of these IRESs are all highly structured. However, while eIF4G is known to be involved in the assembly of 48S ribosomal complex for type I (CA16 and Echo 9) and type II (EMCV) IRESs (Pestova et al., 1996a,b; Sweeney et al., 2014) and thus possibly plays a role in recruiting DDX3X onto these IRESs, it is not required for HCV IRES activity (Pestova et al., 1998; Gonzalez-Almela et al., 2018). As a result, the mechanism underlying DDX3X stimulation in HCV IRES activity and the factors that may recruit DDX3X to HCV IRES remain unclear.

DDX3X has already been reported to enhance HCV IRES activity previously (Geissler et al., 2012). However, in this study authors stated that DDX3X, by interacting with eIF3 and the 40S ribosomal subunit, functioned as a general translational factor that promotes the formation of the 80S ribosome complex. On the other hand, several other reports have shown that DDX3X facilitates the initiation of translation of a particular set of mRNAs with a long or structured 5′-UTR (Lai et al., 2010; Soto-Rifo et al., 2012). On these mRNAs, DDX3X is thought to unwind the 5′ RNA secondary structure and increase the ribosome scanning process. Our results showed that the destabilization of domain VI eliminated the necessity of DDX3X for EV71 IRES activity and that DDX3X did not significantly impact cap-dependent translation, thus supporting the latter scenario. It has been reported that the DEAD-box RNA helicases bind to the sugar backbone of the RNA; hence, the DEAD-box RNA helicases show no sequence specificity for RNA binding *in vitro* (Sengoku et al., 2006). This phenomenon was confirmed in our *in vitro* RNA binding assays (**Figure 4**). Nevertheless, our data showed that DDX3X could interact with the truncated eIF4G which binds specifically to domain V. Thus, it is very likely that *in vivo* DDX3X is preferably recruited to domain VI or a region nearby by associating with eIF4G and eIF4A, and exerts its unwinding activity on domain VI.

One may argue that the inhibition of the IRES activity by DDX3X knockdown is actually caused by a secondary effect resulted from DDX3X silencing *in vivo*. While this possibility cannot be completely ruled out by this study, our data support that DDX3X directly participates in the IRES-mediated translation for at least two reasons. First, the blocking of DDX3X by a DDX3X-specific antibody in the *in vitro* translation assays was not expected to induce any secondary reactions in the cell-free extracts; yet, the IRES-mediated translation was still significantly reduced by DDX3X blocking. Second, if the suppression of the IRES activity were due to a secondary effect of DDX3X knockdown, the translational activities of mutant IRESs M2 and M3 would have not been increased simply by destabilizing the secondary structure, but should have been suppressed as much as the wild-type IRES. However, the results of **Figure 5** revealed that DDX3X was dispensable once the secondary structures of domain VI were destabilized. Based on these observations, we believe that DDX3X RNA helicase does have a direct role in the IRES-mediated translation.

In this study, we showed that EV71 infection significantly enhanced the IRES activity (**Figure 2A**). Of all of the viral proteins investigated, 2A^pro^ and 3C^pro^ exhibited the enhancement effect, which was dependent on their protease activities. It is well known that EV71 2A^pro^ cleaves eIF4G, leading to the shut off of cap-dependent translation and stimulation of the viral IRES activity (Krausslich et al., 1987; Hambidge and Sarnow, 1992; Liebig et al., 1993; Borman et al., 1997). However, the mechanisms by which 2A^pro^ enhances IRES-dependent translation may not be limited to just redirecting the translational machinery and supplies to IRES-dependent translation. As Picornaviruses replicate rapidly in the cytoplasm, their translation requires a growing storehouse of cytoplasmic supplies of ITAFs. Interestingly however, many of the ITAFs are nuclear proteins, e.g., PTB, PCBP, SRp20, FBP1, La, UNR, GARS, etc. Thus, Picornaviruses need to evolve strategies to disrupt the nucleus-cytoplasm trafficking at the nuclear pore. Enterovirus 2A^pro^ is reported to cleave at least three nucleoporins—Nup62, Nup98, and Nup153—that alter the traffic between the nucleus and the cytoplasm (Park et al., 2010; Watters and Palmenberg, 2011). Thus, the cytoplasmic storehouse can be restocked with sufficient supplies to promote IRES-dependent translation.

Diverse mechanisms may also account for the enhanced IRES activity mediated by 3C^pro^. In addition to cleaving the viral polyprotein, the 3C^pro^ protein of PV has been reported to cleave eIF5B during a viral infection. The resulting C-terminal fragment of eIF5B can substitute eIF2 as an initiation factor to deliver Met-tRNA_i_^met^ to the 40S ribosome while eIF2-α is phosphorylated during a viral infection (White et al., 2011). Moreover, the PV 3C^pro^ also can cleave G3BP, an endoribonuclease that interacts with RasGAP and facilitates the assembly of stress granules (SGs) in cells exposed to stress (Tourriere et al., 2003). In response to stress, the mRNAs and the associated proteins in the translating ribosomes are rapidly translocated into SGs and translation is inhibited (Mokas et al., 2009). It has been shown that PV infection causes an initial formation of SGs in the cells; however, the viral 3C^pro^ quickly cleaves G3BP and prevents the accumulation of SGs, thus resuming viral RNA translation and replication under an eIF2α-phosphorylated condition (White et al., 2007, 2011). As a result, 3C^pro^ is able to stimulate the IRES-dependent translation in an eIF2-independent manner. Conversely, the 3C^pro^ cleavage products of PTB, a stimulatory ITAF for Picornaviral IRESs, has been shown to suppress IRES activity, which is thought to switch from translation to replication (Back et al., 2002; Perera et al., 2007).

## Conclusion

In conclusion, in this report we have provided evidence demonstrating that the DEAD-box RNA helicase DDX3X employs its RNA helicase activity to resolve the secondary structure near the ribosome entry site, thus increasing the ribosome scanning process. DDX3X is also involved in many viral IRESs, e.g., coxsackievirus, echovirus, EMCV, and HCV, highlighting DDX3X as an important target for the development of antiviral drugs. The Picornavirus-encoded proteases, 2A^pro^ and 3C^pro^, can enhance IRES-mediated translation with their protease activities, which could be due to the redirection of the translational machinery to the IRES-dependent translation and the increment of nuclear ITAFs flowing from the nucleus into the cytoplasm to form a translational complex favorable for viral mRNA translation.

## Author Contributions

L-HH conceived the project and wrote the manuscript. Y-SS, A-HT, Y-FH, S-YH, and Y-CL conducted the experiments.

## Conflict of Interest Statement

The authors declare that the research was conducted in the absence of any commercial or financial relationships that could be construed as a potential conflict of interest.
