# Supplementary figures and images for: Stimulation of the Internal Ribosome Entry Site (IRES)-Dependent Translation of Enterovirus 71 by DDX3X RNA Helicase and Viral 2A and 3C Proteases
Source: Front Microbiol. 2018 Jun 19;9:1324. doi: 10.3389/fmicb.2018.01324 (PMC6018165; doi:10.3389/fmicb.2018.01324)

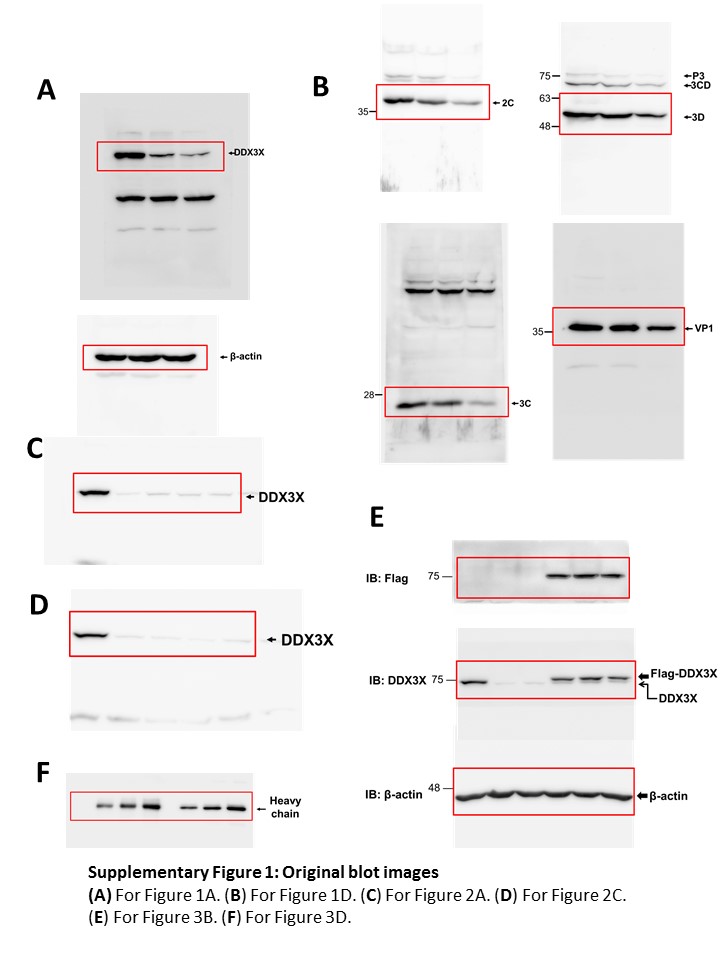

Supplement: Supplementary file 1 [file Image_1.JPEG]

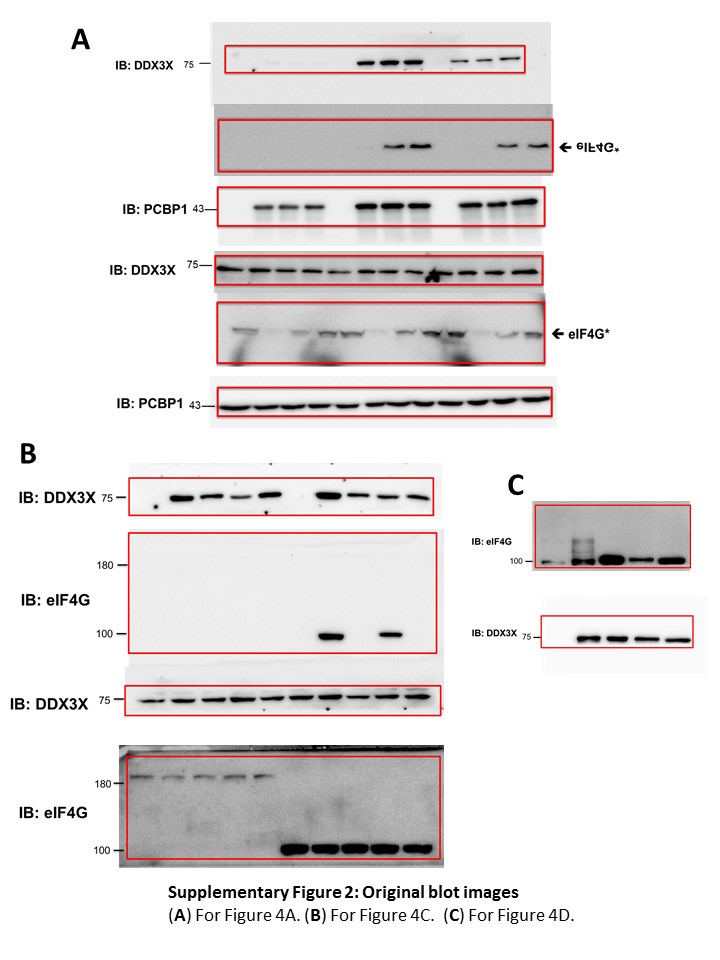

Supplement: Supplementary file 2 [file Image_2.JPEG]

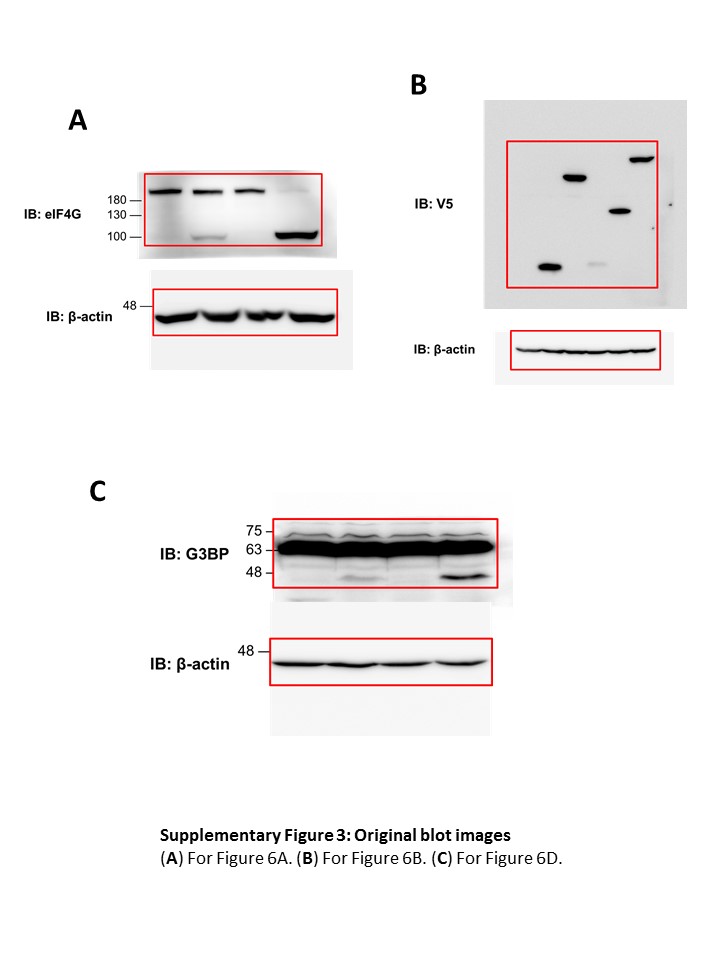

Supplement: Supplementary file 3 [file Image_3.JPEG]
